# Edible Films Based on Plant and Animal Origin Proteins: Comparison of Some Mechanical and Physicochemical Characteristics

**DOI:** 10.1002/fsn3.4712

**Published:** 2025-03-17

**Authors:** Gülistan Okutan, Güneş Koç, Ümran Cansu, Gökhan Boran

**Affiliations:** ^1^ Technical Sciences Vocational School Siirt University Siirt Türkiye; ^2^ Department of Food Engineering Van Yüzüncü Yıl University Van Türkiye; ^3^ Vocational School of Organized Industrial Zone Harran University Şanlıurfa Türkiye

**Keywords:** casein, edible films, gelatin, gluten, mechanic strength, morphology

## Abstract

Edible films were manufactured from three different proteins to evaluate their mechanical strength and some physicochemical features. Wheat gluten (WG), cow hide gelatin (CHG), and cow milk casein (CMC) were used at three different concentrations (5%, 6%, and 7% w/v for WG or 2%, 3%, and 4% w/v for both CHG and CMC) for the film samples. Water activity of the film samples varied within a rather narrow gap, which was between 0.26 and 0.36, with the highest values for WG films and the lowest for CMC. WG and CMC gave basic films while CHG resulted in acidic films with a pH value between 5.6 and 5.7. CHG films showed the highest conductivity while pH and conductivity increased as CHG concentration increased. WG resulted in opaque and dark colored films while CHG and CMC led to almost transparent and light colored films. Water vapor permeability of CMC films was slightly higher compared to CHG and WG counterparts with values around 2.0 × 10^−14^ g m/s Pa m^2^. In addition, tensile strength of CHG films was significantly higher than CMC and WG counterparts with values over 25 N/mm^2^ and more flexible with higher values of Young's modulus and elongation at break. It is concluded that CHG may be utilized by the food industry to manufacture edible films with superior mechanical features along with ease of dissolving and transparent visual characteristics, while WG and CMC might be preferred for more rigid, opaque, and dark colored films as needed.

## Graphical abstract

This manuscript presents valuable data on comparison of mechanical strength and some other physicochemical features of edible films obtained by three different proteins. Gelatin leads to an extendable film with highly elastic structure while gluten and casein films are more rigid but strong. Visual look of the films is also comparable, and gelatin films are transparent and light in color while casein and gluten films are more like yellowish and turbid to some extent. Gelatin is concluded to be a better carrier protein compared to casein and gluten for production of edible films due to its superior mechanical and visual features along with its ease of use, high solubility, and homogeneous film structure achieved.

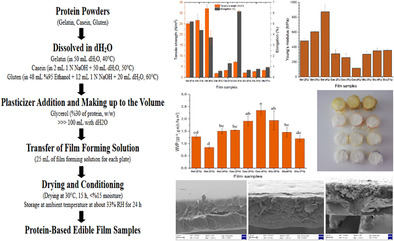

## 1 Introduction

Edible films and coatings have brought a different dimension to the packaging industry by bringing together the concepts of food, packaging, and protection (Kozlu and Elmacı 2021). Increasing consumer awareness, negative perception toward synthetic materials used in packaging industry, concerns on environmental pollution and elevated carbon footprint, and high costs in packaging industry have required the production of new food packages that do not harm the environment, are obtained from natural resources, and provide good protection for food with a small amount of material (Karahan, Meral, and Kılınçeker 2020). Thus, there has been a substantial interest in biodegradable packaging materials and edible films and coatings. Specifically, edible films made from naturally renewable, biodegradable, and edible biopolymers have received increased attention from the researchers and manufacturers in the food and pharmaceuticals industry since the late 1980s. Barrier properties such as moisture, gas, flavor, and lipid permeability of edible films can tremendously improve the food quality (Ge et al. 2018). The main biomaterials used to produce edible films are proteins, lipids, and carbohydrates (Fallah et al. 2021). Among these biopolymers, food proteins are considered promising materials for producing edible active packaging systems due to their high elasticity and plasticity. In addition, protein‐based films have the ability to transport various bioactive components such as antioxidants, antimicrobial agents, flavorings, and colorants, which might be utilized for food preservation and enrichment (Mohammadian et al. 2021). Edible protein films may be formulated with plant‐based proteins (corn zein, wheat gluten, soy protein, pea protein, sunflower protein, peanut protein, and wheat germ protein) or animal‐derived proteins (keratin, collagen, gelatin, fish myofibrillar proteins, egg white proteins, milk casein, and whey proteins) (Dursun and Erkan 2009).

Gelatin is obtained by partial hydrolysis of collagen, which has a favorable film‐forming ability. As it is abundant, it is readily available to be used in production of edible films. Edible gelatin films form effective barriers against UV rays, flavors, and oxygen (Fallah et al. 2021). One of the other widely used protein is gluten. Gluten, a by‐product of starch production, is an interesting raw material for the development of biopolymers due to its unique viscoelastic properties. It is also readily available in large quantities and at low cost. Gluten is used as a biopolymer due to its network formation potential, being relatively inexpensive, abundant, biodegradable, and edible (Sharma et al. 2017). Casein is a mix of milk proteins that may be used as an ingredient in the production of edible films. Casein is soluble and can form films that resist denaturation and/or coagulation even at high temperatures, and therefore its film remains stable over a wide range of temperatures, pH, and salt concentrations. The casein‐based films can be modified as wished depending on manufacturing conditions (Apriliyani et al. 2020).

Despite that all above‐mentioned proteins were previously evaluated for their characteristics in the relevant literature, they have not been yet studied in terms of their potential in edible film manufacturing comparatively. Therefore, in this study, WG, CHG, and CMC were used at different concentrations to assess the effect of protein itself and its concentration on mechanical strength and physical features of the resultant films for evaluation of their potential in edible film manufacturing for the food industry.

## 2 Materials and Methods

### 2.1 Materials

Cow hide gelatin and WG proteins were purchased from Sigma Aldrich (St. Louis, MO, USA). CMC was purchased from ABCR GmbH (Karlsruhe, Germany). Glycerol used as plasticizer was purchased from PanReac AppliChem (Darmstadt, Germany). All other chemical reagents were of analytical grade and obtained from Sigma Aldrich (St. Louis, MO, USA).

### 2.2 Methods

#### 2.2.1 Study Design

A total of nine film samples was produced by three different proteins and at three different concentrations that was 2%, 3%, and 4% (w/v, in dH_2_O) for CHG and CMC; and 5%, 6%, and 7% (w/v, in dH_2_O) for WG. All samples of protein films were prepared, dried, and conditioned in the same manner as described below.

#### 2.2.2 Production of Edible Film Samples

Edible films were obtained according to a previously reported method (Theerawitayaart et al. 2019) with slight modifications. Edible film solutions were prepared by dissolving proteins in varying solvent mixes with distilled water by continuous stirring using a heating magnetic stirrer (Heidolph, Schwabach, Germany) at 375 rpm for about an hour at varying temperatures. Glycerol was used as a plasticizer at a ratio of 30% of protein (w/w) for all samples. Dissolution of CMC was assured by adding 2 mL of 1 N NaOH to about 50 mL of distilled water, which was then completed to 100 mL by distilled water. Dissolution of WG was assured by using 48 mL of 95% ethanol, 12 mL of 1 N NaOH, and 20 mL of distilled water, which was then completed to 100 mL with distilled water. 25 mL of protein solutions were then poured into heat resistant plastic plates (circular, 10.5 cm in diameter) and dried using a conventional circulation oven (Şimşek, Ankara, Türkiye) at 30°C overnight for about 15 h. Dried film sheets were conditioned in a humidity chamber (MgCl_2_ as absorbent achieving about 33% relative humidity) at ambient temperature (22°C ± 2°C) for a day and used for analyses.

#### 2.2.3 Moisture Content

Moisture content of the films were determined according to a previously reported method (Sapper et al. 2018) with slight modifications. Film samples were cut in square pieces in 3 to 3 cm dimensions and dried at 60°C for 24 h in a conventional oven. Moisture content was given by the difference in weight.

#### 2.2.4 Water Vapor Permeability (WVP)

Water vapor permeability were determined according to a previously reported method (ASTM 2014) with slight modifications. Calcium chloride flakes were used as desiccant in a glass desiccator to maintain about 25% relative humidity (RH) at 24°C ± 1°C. The films were placed and sealed at the mouth of hard plastic containers with distilled water inside and kept for 2 days in the desiccator at approximately 24°C ± 1°C and 25% RH. Weight measurements were taken at regular time intervals (approximately 4 h) and decrements in weight of hard plastic containers were plotted as a function of time. Measurements were made in triplicate and mean values were calculated. The linear regression method was used to calculate the slope and the water vapor transmission rate (WVTR) as given by dividing the slope of the line (g/s) by the surface area (m^2^) of the film exposed to permeation of water vapor. After permeability tests, WVP was calculated using the following equation:

(1) $$\mathrm{WVP}\;\left(g.m/\mathrm{Pa}.s.m^{2}\right)=\frac{\text{WVTR}}{S\left(R1-R2\right)}\times d$$

where S is the saturation vapor pressure of water (Pa) at the test temperature (24°C), R1 is relative humidity (RH) in the hard plastic container, R2 is RH in the desiccator, and *d* is the thickness of the film sample (m).

#### 2.2.5 pH and Conductivity

pH and conductivity of film forming solutions were determined at ambient temperature by use of a multi‐meter (SG7, Mettler Toledo, OH, USA). About 20 mL of film forming solutions obtained from CHG (2%, 3%, and 4%, w/v), CMC (2%, 3%, and 4%, w/v), and WG (5%, 6%, and 7%, w/v) were used for pH and conductivity measurements after calibration with pH buffers and conductivity standards.

#### 2.2.6 Opacity and Transparency

Opacity and transparency measurement in protein films were carried out according to the method previously reported (Uranga et al. 2019). For this purpose, absorbance (A) and transmittance (T) values of the film samples were measured at 600 nm wavelength using a UV–VIS spectrophotometer (UV1800, BIOBASE, Shandong, China), and opacity and transparency values were calculated by normalizing with film thickness according to the following formulae:

(2) $$\text{Opacity}=A_{600}/\text{Film thickness}\;\left(\mathrm{mm}\right)$$

(3) $$\text{Transparency}=T_{600}/\text{Film thickness}\;\left(\mathrm{mm}\right)$$

#### 2.2.7 Color Parameters

Color parameters were determined by a portable colorimeter (CSM5, PCE Instruments, Southampton Hampshire, UK) both in film samples and film solutions. “*L*” indicates darkness to lightness with values between 0 and 100, “*a*” indicates greenness to redness with values between −128 and +127, and “*b*” indicates blueness to yellowness with the same range of values as in *a*. Total color difference (ΔE) was calculated based on the difference between *L*, *a*, and *b* values of the film samples and a dozen of A4 white print papers stacked on each other. Whiteness index (WI) was also calculated based on the *L*, *a*, and *b* values of the film samples according to the formulae below:

(4) $$\mathrm{\Delta E}=\sqrt{{\left(\mathrm{\Delta L}\right)}^{2}+{\left(\mathrm{\Delta a}\right)}^{2}+{\left(\mathrm{\Delta b}\right)}^{2}}$$

(5) $$\mathrm{WI}=100-\sqrt{{\left(100-L\right)}^{2}+a^{2}+b^{2}}$$

#### 2.2.8 Viscosity and Temperature Sweep

Viscosity of film forming solutions was investigated as a function of protein concentration and temperature. Film forming solutions of each protein sample were prepared at concentrations of 1%, 2%, and 5% (w/v) and used for viscosity measurements at 25°C and 30°C. For this purpose, a computer controlled rotational rheometer (DVIII Ultra, Brookfield Inc., MA, USA) equipped with small sample adapter and a circulation water bath was used (GMIA 1986). Temperature sweep test was also performed for film forming solutions only at 5% (w/v) concentration while temperature of the samples was changed from 50°C to 5°C and back again to 50°C to determine if a sol–gel transition or a dramatic change in viscosity occur in samples depending on temperature. Viscosity of these protein solutions was recorded at 10 s intervals during the test (Kołodziejska et al. 2008).

#### 2.2.9 Mechanical Properties

Tensile strength (TS, MPa or N/mm^2^), elongation at break (EB, %), and Young's modulus (YM, MPa) were determined according to the method previously reported (Shi et al. 2019). For this purpose, protein film samples were cut in 10 mm width to 60 mm length and assessed by use of a texture analyzer (TA‐XT2 Plus, Texture Technologies, MA, USA), which is equipped with an A/TG elongation probe. The initial jaw opening was set at 40 mm and the jaw movement speed was 0.5 mm/s. The test was carried out on dry and conditioned films. Tensile strength, elongation at brake, and Young's modulus were calculated according to the following formulae:

(6) $$\mathrm{TS}=\frac{\text{Maximum force}\;\left(N\right)}{\text{Cross section area of the film}\;\left({\mathrm{mm}}^{2}\right)}$$

(7) $$\mathrm{EB}=\frac{L-L_{0}}{L_{0}}\times 100$$

(8) $$\mathrm{YM}=\frac{F}{A}\times \frac{L_{0}}{\mathrm{\Delta L}}$$

where *L*_0_ is the initial length of the film (m), *L* is the length at break (m), *F* is the tension applied to the film (N), *A* is the cross section area of the film (m^2^), and Δ*L* is elongation of the film until the break (m), that is, *L*−*L*_0_.

#### 2.2.10 Morphological Analyzes

SEM images were taken to examine surface and cross section of the films. After the film samples were kept at ambient temperature for 24 h, they were coated with gold and examined under an acceleration voltage of 10 kV (Picchio et al. 2018).

#### 2.2.11 Statistical Analysis

The data obtained were evaluated using JMP 8.0 (SAS, NC, USA) statistical program. The data were evaluated by ANOVA to find out if there was a significant difference among the means and by Tukey–Kramer test for determination of the pairs that were significantly different from each other at a 95% significance level. All measurements were in triplicate at least.

## 3 Result and Discussion

### 3.1 Characteristics of Protein Solutions

#### 3.1.1 pH and Conductivity

Conductivity and pH have an impact on film forming ability of proteins at a certain level, and therefore, it is important to assess these parameters along with other mechanical and visual parameters, which are essential in terms of strength, usability, and compatibility of the resultant edible films (Li et al. 2023). The pH and conductivity values of film forming solutions are given in Table 1. It was observed that the lowest pH value was in CHG solutions and the concentration of CHG caused no significant change in pH values. The ionizable R groups of amino acids play a significant role on pH value. The degree of acidity and basicity is also influenced by amino acid composition (Damodaran 2007). In this context, when protein is composed of highly basic amino acids like lysine, arginine, and histidine, pH value of its solution would be higher than 7 (basic), and in case of acidic amino acids like aspartic and glutamic acid, its pH would be lower than 7 (acidic) (Soga and Heiger 2000). Thus, the differences in pH value of protein solutions studied are due to amino acid profiles of the proteins used in the formulation. For WG and CMC solutions, NaOH was used to dissolve these proteins, which might have probably contributed to high pH values measured for these protein solutions.

**TABLE 1:** pH and conductivity of film forming protein solutions.

Film proteins	Concentration (%)	pH	Conductivity (mS/cm)
CHG	2	5.62 ± 0.02^h^	204.00 ± 0.00^c^
	3	5.67 ± 0.00^gh^	263.70 ± 1.53^b^
	4	5.69 ± 0.01^g^	369.00 ± 3.61^a^
CMC	2	9.94 ± 0.01^e^	3.25 ± 0.01^d^
	3	10.75 ± 0.00^d^	3.58 ± 0.01^d^
	4	9.83 ± 0.00^f^	3.77 ± 0.01^d^
WG	5	13.06 ± 0.01^a^	5.14 ± 0.01^d^
	6	12.93 ± 0.01^b^	4.47 ± 0.03^d^
	7	12.80 ± 0.01^c^	4.34 ± 0.01^d^

*Note:* Significance level for the data presented (a, b, c, d, e, f, g, h) is *p* < 0.05.

The conductivity of film forming solutions changed significantly among the samples. CHG solutions showed higher conductivity compared to that of other two proteins, which was due to higher amount of ions accumulated when CHG is dissolved in water. The amount of ions accumulated in a protein solution highly depends on protein solubility and its amino acid profile, greatly affecting electricity and conductivity (Lepetit et al. 2002). Furthermore, protein concentration is also considered to be a significant factor affecting the electrical conductivity (Songchotikunpan, Tattiyakul, and Supaphol 2008; Ratanavaraporn et al. 2010). Therefore, higher concentrations of CHG and CMC samples might have caused higher conductivity levels as observed (Table 1).

#### 3.1.2 Viscosity and Temperature Sweep

**FIGURE 1:**
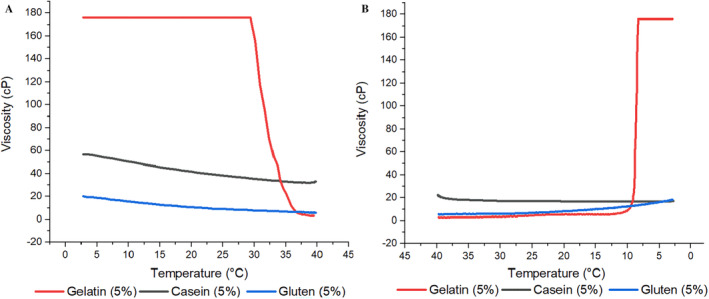
Viscosity vs. temperature (A: Heating and B: Cooling) in film forming solutions of gelatin, casein and gluten.

Apparent viscosity is a term used to accurately describe the resistance to flow in the case of protein systems as many of these systems are non‐Newtonian, with changing viscosity as a function of shear rate. In order to examine the viscosity of three different protein solutions depending on the temperature, 5% protein solutions were prepared, and the viscosity was followed during heating from 0°C to 40°C and cooling back from 40°C to 0°C. Figure 1A,B shows the viscosity vs. temperature during heating and cooling, respectively. It was observed that CHG solution had a higher apparent viscosity than CMC and WG counterparts. In addition, viscosity of CHG solution showed dramatic changes during heating and cooling, that was for gel–sol and sol–gel transition, respectively, while there was no such transition in other two protein samples. For CMC and WG solutions, apparent viscosity was either not affected or only changed slightly compared to CHG sample. Difference in this behavior is because average molecular weight and molecular structure of the proteins are different, leading to difference in protein–solvent interactions and network forming capacity of protein fibers (Beliciu and Moraru 2011). Viscosity sharply increased by cooling in CHG sample, which was mainly attributed to the role of glycine and proline residues in renaturation and initial stages of network formation (Djabourov, Lechaire, and Gaill 1993). In fact, these sharp viscosity changes were characteristic in CHG solutions while heating and cooling (Gómez‐Guillén et al. 2011).

### 3.2 Characteristics of Protein Films

#### 3.2.1 Moisture Content and a_w_ Values of Film Samples

Moisture content and a_W_ values of edible films play a critical role, influencing not only microbial safety but also mechanical strength and flexibility. Consequently, these factors can significantly impact the shelf life of packaged materials. Table 2 shows the moisture content of film samples prepared with CMC, CHG, and WG at different concentrations and a_W_ values of the films are also presented (Table 2). Statistical analysis showed that moisture content of the films were significantly (*p* < 0.05) different from each other and similarly changed significantly depending on concentration of the proteins. That is most probably due to differences in protein structure, which may lead to varying amount of water absorbed, binding strength, and hydrophilicity of the proteins that may result in different rates of moisture loss during dehydration (Denavi et al. 2009). It was observed that a_W_ was very similar in all film samples and changed within a rather narrow range of 0.30–0.35, 0.26–0.28, and 0.30–0.36 for CHG, CMC, and WG samples, respectively. However, it was determined that a_W_ values of CMC films were slightly lower than that of the others despite of the highest moisture content. That is probably because of CMC itself being more hydrophilic when dissolved, leading to retention of more water, which eventually result in lower a_W_ values but higher moisture content. The greater water absorption capacity in CMC films may be attributed to their stronger hydrophilic characteristics. Glycerol may have also been a factor loosening microstructure of the blended films but also increasing the hydrophilic interactions at different ratios by exposing the hydroxyl groups (Su et al. 2010).

**TABLE 2:** Moisture content and a_W_ values of film samples.

Film proteins	Concentration (%)	a_W_ (sheet)	a_W_ (powder)	a_W_ (solution)	Moisture (%)
CHG	2	0.35 ± 0.01^a^	0.30 ± 0.01^ab^	0.84 ± 0.00^c^	10.67 ± 0.27^c^
	3	0.30 ± 0.00^c^	0.31 ± 0.00^ab^	0.84 ± 0.00^c^	12.09 ± 0.49^bc^
	4	0.30 ± 0.00^c^	0.31 ± 0.01^ab^	0.84 ± 0.00^c^	11.34 ± 1.17^bc^
CMC	2	0.26 ± 0.00^d^	0.28 ± 0.01^ab^	0.93 ± 0.00^a^	17.65 ± 1.88^a^
	3	0.26 ± 0.00^d^	0.27 ± 0.00^b^	0.93 ± 0.00^a^	17.00 ± 0.14^a^
	4	0.27 ± 0.00^d^	0.27 ± 0.00^b^	0.93 ± 0.00^a^	16.65 ± 0.66^a^
WG	5	0.36 ± 0.01^a^	0.31 ± 0.00^ab^	0.86 ± 0.00^bc^	14.81 ± 0.37^ab^
	6	0.32 ± 0.01^b^	0.31 ± 0.00^ab^	0.91 ± 0.00^ab^	10.39 ± 0.98^c^
	7	0.30 ± 0.00^c^	0.33 ± 0.00^a^	0.90 ± 0.00^abc^	13.95 ± 0.41^abc^

*Note:* Significance level for the data presented (a, b, c, d, e, f, g, h) is *p* < 0.05.

#### 3.2.2 Color of Film Samples

Color attributes of edible films are of utmost importance as they directly influence consumer acceptability (Chandra Lal, Yambrach, and McProud 2015). In general, edible films should strive to be as colorless as possible, not interfering with the material in it (Debeaufort, Quezada‐Gallo, and Voilley 1998). However, role of the light in oxidation is crucial especially for high lipid and protein products, and therefore, light barrier ability of a packaging film is a core function to protect light‐sensitive foods (Gontard, Guilbert, and Cuq 1992). Table 3 presents the results of the color evaluation of the film samples studied. CHG films exhibited significant variations in color, with the lightest appearance indicated by the highest *L* and WI values, while WG and CMC films were relatively darker. The high WI of CHG films is a desirable attribute for successful applications in various food products. It should be noted that CHG played a significant role in producing almost colorless and transparent films (Tulamandi et al. 2016). *L* value did not show significant changes among the different concentrations of film samples, whereas *a* and *b* values differed significantly for CMC and WG films. Hence, these findings suggest that CMC and WG films exhibit a yellow‐brown color compared to CHG films. The total color difference (∆E) index, which is influenced by *L*, *a*, and *b* values, can indicate the difference between the color of items in the color space (Rao et al. 2010). The changes in film concentration affected the *L*, *a*, and *b* values, resulting in alterations in the ∆E values of the films. The ∆E indices of CMC and WG films observed in this study were higher compared to CHG films due to higher differences in the *L*, *a*, and *b* values. There are different studies in the relevant literature reporting color values of edible films manufactured by various carrier materials. Incorporation of bioactive ingredients into edible films also affects the visual characteristics of the resultant films depending on its concentration, solubility, and processing (Shahbazi et al. 2021).

**TABLE 3:** Color parameters of film samples.

Proteins	Concentration (%)	*L*	*a*	*b*	∆E	WI
CHG	2	96.51 ± 0.63^a^	4.09 ± 0.30^e^	−2.12 ± 1.50^f^	4.63 ± 0.21^e^	94.04 ± 0.38^a^
	3	95.79 ± 0.73^a^	3.43 ± 0.83^f^	−0.38 ± 1.28^f^	4.90 ± 0.30^e^	94.34 ± 0.08^a^
	4	93.49 ± 0.77^a^	0.98 ± 0.27^g^	3.69 ± 1.23^e^	7.71 ± 1.20^e^	92.40 ± 1.07^a^
CMC	2	85.54 ± 1.64^b^	3.42 ± 0.51^f^	20.14 ± 0.84^d^	25.25 ± 1.32^d^	74.95 ± 1.69^b^
	3	82.51 ± 0.83^bc^	5.28 ± 0.24^c^	22.80 ± 1.15^cd^	28.88 ± 1.71^cd^	70.78 ± 1.36^bc^
	4	79.36 ± 1.66^c^	6.65 ± 0.72^a^	23.88 ± 1.71^c^	31.15 ± 2.17^c^	67.98 ± 2.26^cd^
WG	5	80.21 ± 1.96^c^	4.81 ± 0.81^d^	29.23 ± 2.42^b^	35.66 ± 2.80^b^	64.34 ± 2.91^de^
	6	79.36 ± 1.66^c^	4.84 ± 0.37^d^	33.76 ± 3.03^a^	40.16 ± 3.41^a^	60.13 ± 3.44^e^
	7	78.94 ± 0.77^c^	5.78 ± 0.49^b^	29.94 ± 1.15^b^	36.84 ± 1.36^ab^	62.93 ± 1.39^de^

*Note:* Significance level for the data presented (a, b, c, d, e, f, g, h) is *p* < 0.05.

#### 3.2.3 Water Vapor Permeability (WVP)

**FIGURE 2:**
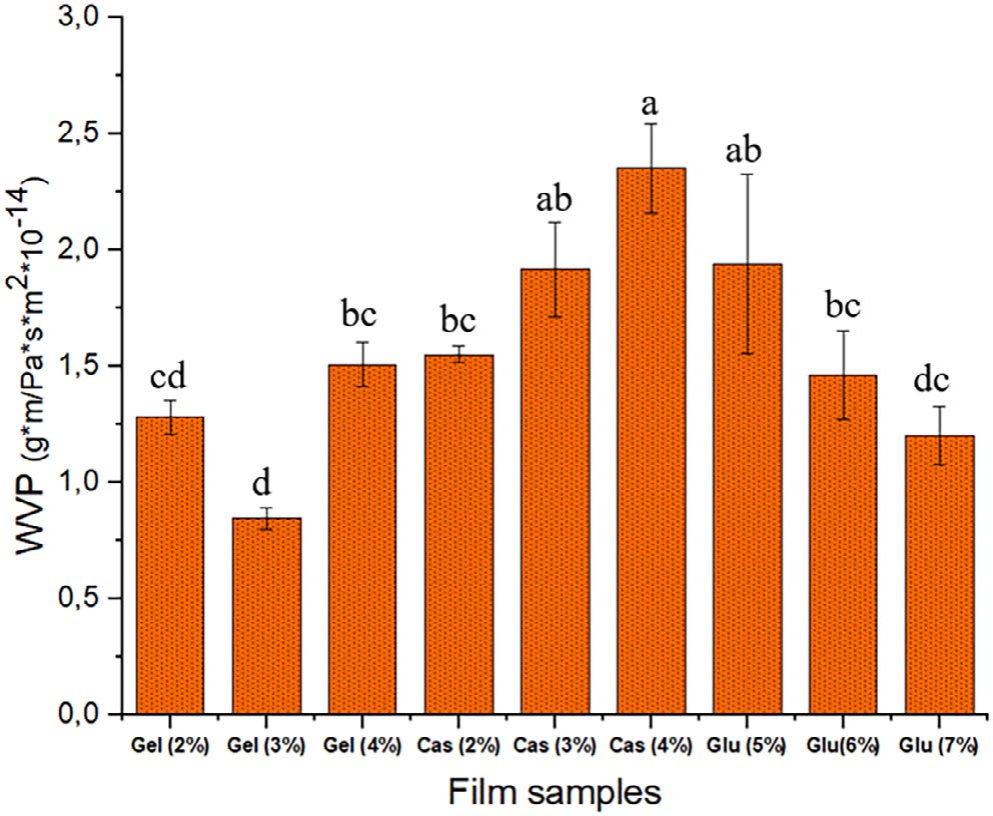
Water vapor permeability of protein films.

Water vapor permeability is a significant parameter for packaging materials as it represents the permeability of not only water vapor but also air in general, in and out through the packaging material during handling and storage of the products covered within, which greatly affect the shelf life, safety and appearance (Chandra Lal, Yambrach, and McProud 2015; Debeaufort, Quezada‐Gallo, and Voilley 1998). Figure 2 illustrates the water vapor permeability values of the film samples at varying concentrations. The increase in protein concentration led to varying degrees of increments in water vapor permeability in CHG and CMC films on the contrary of WG films. The differences in water vapor permeability between these protein films were more noticeable and higher in CMC films. The increased WVP may be attributed to enhanced interactions between the protein chains, resulting in a compact structure. It has been reported that WVP of protein films depends on the protein network structure and the relationship of the film constituents with water (Ansorena, Zubeldía, and Marcovich 2016). Furthermore, the protein concentration in the structure contributes to water permeability as it engages in hydrogen bonding with both free water entrapped within the film and with the protein itself. These interactions enhance the inter‐chain space of CHG and CMC polymers, thereby increasing the mobility of water molecules in the film matrix and improving water migration through the film. These findings are also supported by the higher TS (tensile strength) in CHG and CMC films, which are associated with the formation of a stronger network. Water vapor transfer typically occurs through the hydrophilic portion of the film network, depending on the hydrophilic/hydrophobic ratio of the film constituents (Tongnuanchan, Benjakul, and Prodpran 2012). Among the three proteins, WG films exhibited decreasing WVP values with increasing protein concentration, which may be attributed to higher protein concentration compared to other two protein films. This may be because of increased intermolecular hydrogen bonding, resulting in lower water absorption by WG films (Kaewprachu et al. 2016).

#### 3.2.4 Opacity and Transparency

Thickness of WG films increased with increasing concentration, ranging from 0.2 to 0.4 mm. However, there was no difference in thickness observed for CHG and CMC films at different concentrations, remaining at 0.2 mm (data not shown). Identical film thickness in CHG and CMC films may be attributed to the interaction of protein molecules, leading to a higher degree of compaction in the film matrix (Tongnuanchan et al. 2015). The increase in film thickness of WG films can be attributed to the higher solid content or the inability of peptide chains in WG to form a compact film network at higher concentrations, which also causes opacity (Ahmad et al. 2012). Opacity, which is often falsely used with turbidity interchangeably, is a measure to what extent a material can block the light transmission. Transparency, on the other hand, is the opposite and is a measure to what extent the light can go through. The degree of homogeneity in a film determines its transparency, which is influenced by the film's formulation and production techniques (Friesen, Chang, and Nickerson 2015). Optical properties such as opacity and transparency, are essential sensory aspects for edible films and coatings to be accepted by consumers (Galus and Kadzinska 2016).

**FIGURE 3:**
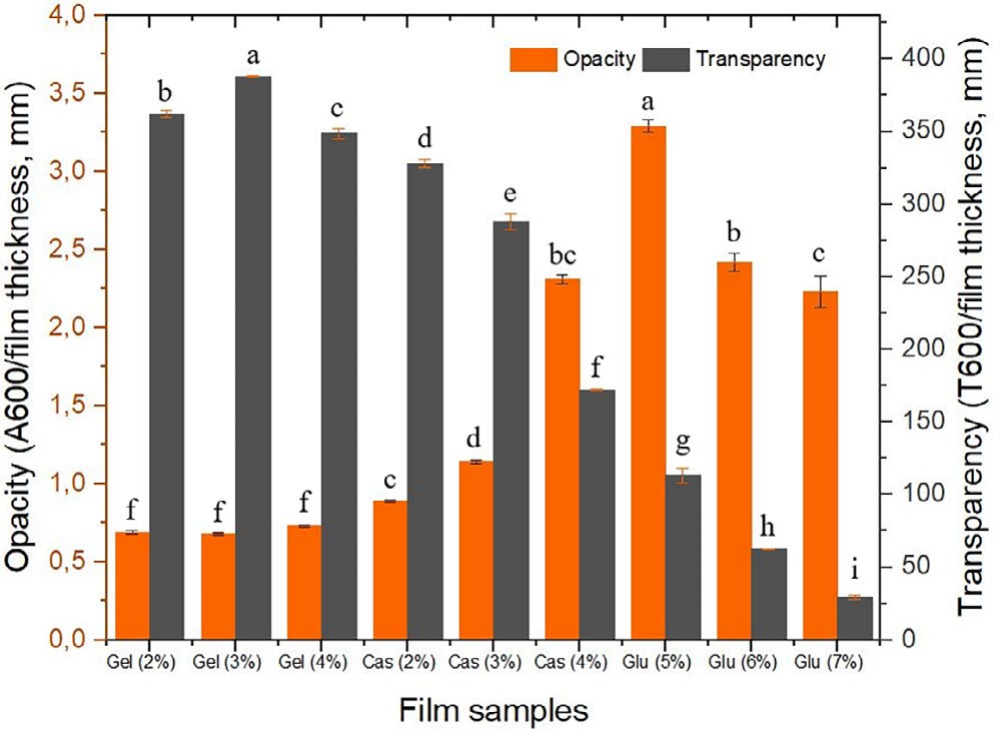
Opacity and transparency of protein films.

Figure 3 illustrates opacity of the films produced from CHG, CMC, and WG. A one‐way analysis of variance revealed no significant difference in opacity among the CHG films prepared at different concentrations. Opacity of CHG films was consistently low, ranging from 0.70 to 0.74, with no significant difference among the concentrations. Conversely, opacity increased from 0.9 to 2.3 with increasing concentration in CMC films, while it decreased from 3.3 to 2.2 in WG films as the concentration increased. Pronounced opacity found in CMC and WG films may be likely due to three‐dimensional globular structure of the proteins. Globular proteins tend to deflect more light than fibrous proteins, resulting in more opaque and darker films. In contrast, CHG contains fibrous collagen fractions, resulting in less opaque and more transparent films. The transparency of films is inversely proportional to their opacity.

Transparency of edible films is a desirable feature in many food coating applications as it allows customers to examine the products before purchasing. On the other hand, the coating material must also shield the food from the detrimental effects of the light. Therefore, high transparency as well as very low transparency may limit the applicability of the films in the food industry (Kowalczyk and Baraniak 2011). As protein content increased, the optical transmittance values of CMC and WG films decreased significantly. Higher transparency in CHG films could also be attributed to the high surface charge on the proteins (Shevkani et al. 2014).

#### 3.2.5 Tensile Strength, Elongation Limit and Young's Modulus

**FIGURE 4:**
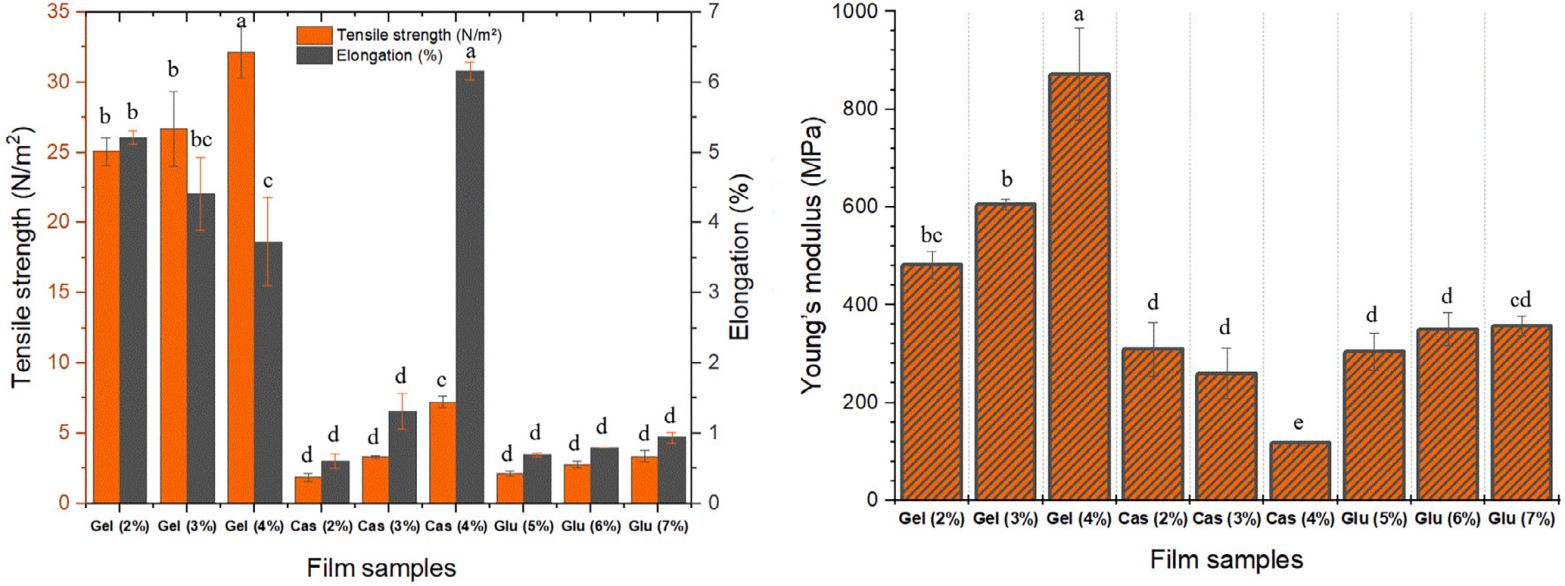
Tensile strength and Young's modulus of protein films.

Packaging materials rely heavily on their mechanical characteristics, including strength, flexibility, and durability, to fulfill their primary purpose (Chandra Lal, Yambrach, and McProud 2015; Debeaufort, Quezada‐Gallo, and Voilley 1998). Figure 4 presents the tensile strength, elongation limit, and Young's modulus of the protein films. The results indicate that tensile strength and elongation limit values were significantly affected by the concentration and the proteins studied. Generally, an increase in protein concentration leaded to an increase in tensile strength. The same was true for elongation limit values of CMC and WG films but not in CHG films. CHG films exhibited decreasing values in elongation limit at higher protein concentrations, indicating that a more rigid but stronger film was obtained. CHG films exhibited significantly higher values in all mechanical parameters compared to CMC and WG counterparts (Fabra, Talens, and Chiralt 2008). CHG films exhibited higher tensile strength due to intermolecular cross‐linking of gelatin molecules and a network formation because of the fibrous structure of gelatin, in contrast to CMC and WG films as these proteins possess globular structure. Gelatin's linear structure and limited monomer composition lead to excellent film‐forming properties. It has also been reported that films based on linear proteins possess higher strength compared to those based on globular proteins (Tulamandi et al. 2016). However, increased protein concentration leads to lower elongation limit for CHG films, while higher for CMC and WG films. Nevertheless, even with this elevation in elongation limit, CMC and WG films remained well below CHG films, except for 4% CMC film. This suggests that more flexible and stretchable films can be produced from 4% CMC films. Increased protein concentration in CHG films led to improved flexibility, resistance to traction, and higher Young's modulus, along with significantly better elongation. Elongation limit values obtained in this study were low in general, but tensile strength and Young's modulus values were in good agreement with the relevant literature (Eghbalian et al. 2021).

#### 3.2.6 Morphological Characteristics of the Films

**FIGURE 5:**
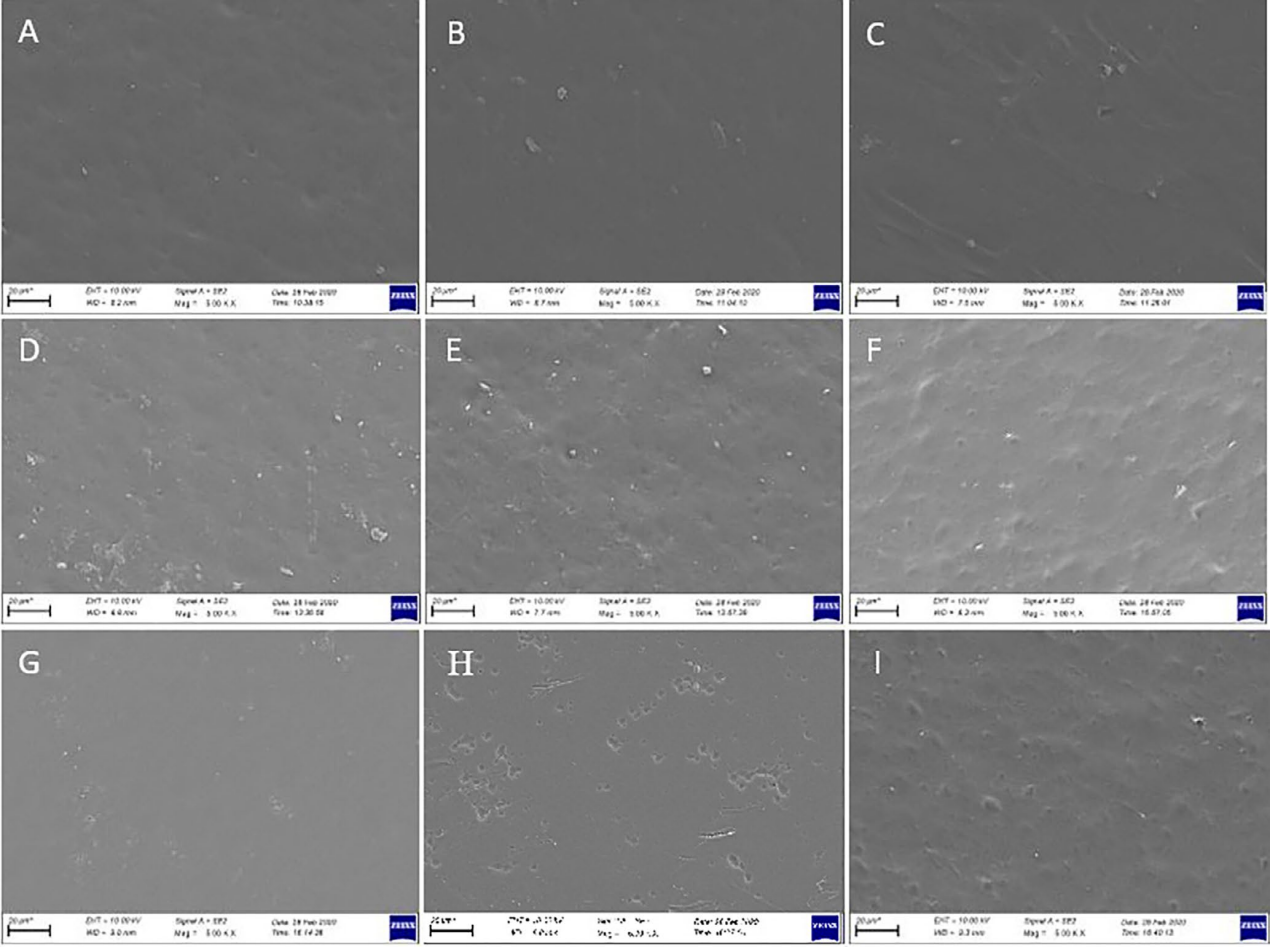
Surface morphology of film samples. (A–C: Films prepared with CHG at concentrations of 2%, 3%, and 4%). (D–F: Films prepared with CMC at concentrations of 2%, 3%, and 4%). (G–I: Films prepared with WG at concentrations of 5%, 6%, and 7%). Magnification is 5.00 K × and the bar below each figure is 20 μm for all samples.

**FIGURE 6:**
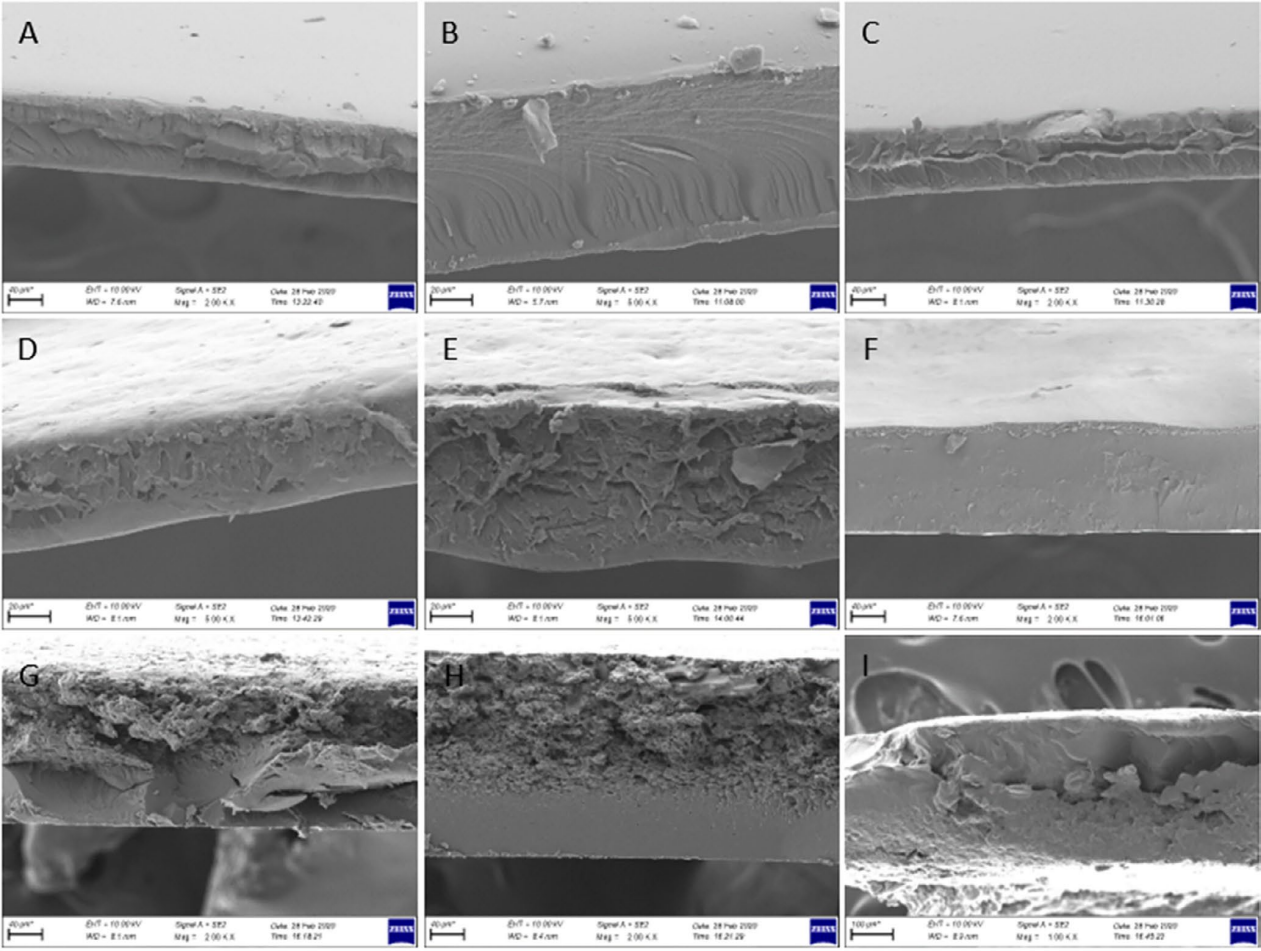
Cross‐sectional morphology of film samples. (A–C: Films prepared with CHG at concentrations of 2%, 3%, and 4%). (D–F: Films prepared with CMC at concentrations of 2%, 3%, and 4%). (G–I: Films prepared with WG at concentrations of 5%, 6%, and 7%). Magnification is 1.00 K × for sample I; 2.00 K × for samples A, C, F, G, and H; 5.00 K × for samples B, D, and E; and the bar below figures is 20 μm for samples B, D, and E; 40 μm for samples A, C, F, G, and H; 100 μm for sample I.

Films samples prepared with CHG at different concentrations exhibited smoother structure as can be seen from both surface and cross‐sectional images (Figures 5 and 6). No voids or fractures were observed in the surface and cross‐sectional images of the films prepared with commercial bovine gelatin. This observation is associated with the fact that gelatin forms a uniform network structure with thin and densely packed protein fibers (Kittiphattanabawon et al. 2016). As previously reported, proper bonding of protein fractions leads to a wellformed gel network and a uniform structure (Benjakul et al. 2009). Smooth network structure and absence of air voids in CHG films give their resistance to the breakage (Kittiphattanabawon et al. 2016). Tensile tests performed on these films revealed their elastic nature and high elongation limit (Figure 4).

Cross‐sectional images of CMC films were examined and it was observed that increase in CMC concentration improved the structure of films. Some air voids and fractures were visible in the cross‐section of 2% and 3% CMC films, while 4% CMC film was quite smooth with almost no void and fractures. The smooth structure observed in 4% CMC film can be attributed to its higher flexibility and tensile strength (Chambi and Grosso 2006). SEM images provide some insight for expectations on tensile resistance of the film samples. It was observed that elongation limit of 4% film was significantly higher compared to that of 2% and 3% CMC films. This was no different for WG films as the presence of air voids and irregular structure, as seen especially in cross‐sectional images of WG films, led to rigid and not flexible films and also an irregular film formation (Jansens et al. 2013). Essentially, voids and sharp cracks in WG films can act as stress collectors, showing premature fractures (Gallstedt et al. 2004). This is further supported by tensile test that led to considerably lower elongation limit values compared to other films, indicating their brittle and rigid nature (Figure 4).

## 4 Conclusions

This study showed that CHG can be successfully used in edible film applications for the food industry due to its high solubility in water, superior film forming ability, and desired visual and mechanical features compared to CMC and WG counterparts. CHG films were less opaque and more transparent compared to the other films studied. CHG exhibited stronger and more flexible films due to its fibrous structure forming a good network. In addition, CHG films were almost perfectly transparent, which may be desired for some specific food products where the visibility of the content is required. Furthermore, CHG films were light in color, which is also desired to avoid any interference originated from the package material on color of the product itself. On the other hand, dark color and low light transmission of CMC and WG films may be useful for those food products, which are very sensitive to oxidation accelerated by light exposure. However, their low flexibility and extensibility are serious concerns that need to be addressed. In addition, WG films exhibited lower water vapor permeability compared to CHG and CMC films. Low water vapor permeability is desirable in packaging materials to prevent water loss from the product or potential contamination of the volatiles from surrounding of the packaged good. Therefore, gelatin (CHG) is concluded to be a better carrier polymer to be used in edible films compared to gluten (WG) and casein (CMC) due to stronger and more flexible films as well as high transparency, which may be desirable for some food products where visibility of the content is required. Thus, the results presented in this study provide useful insights for future studies in the field of edible films for various food products.

## Author Contributions

**Gülistan Okutan:** data curation (equal), formal analysis (equal), investigation (equal), methodology (equal), writing – original draft (equal). **Güneş Koç:** formal analysis (equal), investigation (equal), methodology (equal), writing – original draft (equal). **Ümran Cansu:** data curation (equal), methodology (equal), software (equal), visualization (equal), writing – original draft (equal). **Gökhan Boran:** conceptualization (lead), data curation (equal), funding acquisition (lead), investigation (equal), methodology (equal), project administration (lead), resources (lead), software (equal), supervision (lead), visualization (equal), writing – review and editing (lead).

## Conflicts of Interest

The authors declare no conflicts of interest.

## Data Availability

The data that support the findings of this study are available from the corresponding author upon reasonable request.
